# First stage psychometric testing of a new instrument for adolescents with visual impairment: the Participation and Activity Inventory for Children and Youth (PAI-CY) 13–17 years

**DOI:** 10.1186/s41687-020-00228-3

**Published:** 2020-07-22

**Authors:** Ellen B. M. Elsman, Ruth M. A. van Nispen, Gerardus H. M. B. van Rens

**Affiliations:** 1 Amsterdam UMC, Vrije Universiteit Amsterdam, Ophthalmology, Amsterdam Public Health Research Institute, location VUmc, PK4X 187, De Boelelaan 1117, PO Box 7057, 1007 MB Amsterdam, The Netherlands; 2 Elkerliek Hospital, Ophthalmology, Wesselmanlaan 25, 5707 HA Helmond, The Netherlands

**Keywords:** Visual impairment, Questionnaire, Participation, Psychometric properties, Adolescents

## Abstract

**Background:**

To assess participation of children with visual impairment, the Participation and Activity Inventory for Children and Youth (PAI-CY) was recently developed. This study assessed some initial psychometric properties of the PAI-CY 13–17 years version, and investigated its feasibility.

**Methods:**

Adolescents with visual impairment and their parents (*n* = 72 dyads) completed the self-report and proxy-report version of the 58-item PAI-CY, an evaluation form and several questionnaires measuring related constructs. Item deletion was informed by item responses, inter-item correlations, test-retest reliability, adolescent-parent agreement and participants’ feedback. Known-group validity and concurrent validity with related questionnaires were investigated for the final item-set.

**Results:**

Twelve items had > 20% missing values, whereas 39 items showed floor effects. Eight item pairs showed high inter-item correlations. Test-retest reliability was acceptable for most items (kappa ≥0.4). Evaluation forms showed that over 90% of respondents was neutral to very positive regarding several feasibility aspects such as administration time and comprehensiveness. Adolescent-parent agreement was mostly low. These results informed the deletion of three items. Known-group validity seemed adequate since PAI-CY scores were significantly worse for participants with comorbidity compared to those without. A trend towards worse scores for participants with more severe visual impairment was also observed. Correlations between the PAI-CY and related questionnaires confirmed concurrent validity.

**Conclusions:**

Initial psychometric properties of the PAI-CY 13–17 were acceptable, although more work is needed to assess other psychometric properties, such as the underlying construct. Following implementation in low vision care to assess participation needs, enabling larger samples, acceptability of the PAI-CY 13–17 to end-users should be carefully monitored, especially if alterations are made based on the current study.

## Background

Patient-reported outcome measures (PROMs) evaluate outcomes of health, illness or treatment that are important to patients and directly reported by patients themselves. They include for example symptom severity, health perception, and quality of life [1]. PROMs are increasingly used for evaluating health services, establishing treatment effectiveness, and informing clinical decision-making. PROMs can contribute to clinician-patient communication, facilitate the process of shared decision-making and improve patient satisfaction with care [2–4].

Numerous PROMs currently exist, and a fair share are developed specifically for children. These include generic instruments (e.g. [5–7]) facilitating comparison of different patient populations, as well as disease-specific instruments specifically targeted to those with the condition of interest. Until recently there was a paucity in the development of PROMs to capture the perspectives of children and adolescents with visual impairment (VI), possibly relating to the challenges to develop PROMs for this population. For instance, psychometric validation of PROMs depends on large and representative samples, but the population of children with VI is numerically small, complex, heterogeneous and difficult to reach. Despite the challenges, various instruments for children and adolescents with VI have been developed in recent years, mainly focusing on vision-related quality of life and functional vision (e.g. [8–12]).

We recently developed the Participation and Activity Inventory for Children and Youth (PAI-CY), which focuses on participation of children with VI. Four age-appropriate versions of the PAI-CY were developed reflecting the developmental age-bands of the World Health Organization (WHO): 0–2 years (proxy-report), 3–6 years (proxy-report), 7–12 years (proxy and self-report) and 13–17 years (proxy and self-report). Additionally, the PAI Young Adults (PAI-YA) was developed aimed at persons aged 18–25 years. The PAI-CY was developed to structure the process of identifying needs from the perspectives of children with VI and their parents at Dutch low vision services. Its content was shaped involving end-users as stakeholders in a concept-mapping study [13], ensuring face and content validity, and a small-scale pilot-study demonstrated its feasibility and acceptability [14].

This study reports the first stage psychometric evaluation of the PAI-CY 13–17. This includes the assessment of some important psychometric properties, such as test-retest reliability, known-group validity and concurrent validity with other instruments. These analyses were intended to capture the worst performing items, resulting in initial item reduction. Moreover, some feasibility aspects were assessed.

## Methods

### Subjects and procedures

Adolescents aged 13–17 years registered at two Dutch low vision services and their parents/caretakers (parents for brevity) were invited to participate. Participants had to have adequate knowledge and understanding of the Dutch language. Children with major cognitive impairment were excluded from invitation by the low vision rehabilitation centers. Prior to participation in the study, written informed consent was obtained from adolescents and their parents. Adolescents completed the questionnaires through a face-to-face interview in their own homes, whereas parents completed the questionnaires through a web-based survey. For adolescents, the questionnaires consisted of the PAI-CY 13–17, a self-constructed evaluation form to further assess some feasibility aspects, and the Dutch versions of the Child and Adolescent Scale of Participation (CASP) [7], Kidscreen-27 [5], and the Functional Vision Questionnaire for Children and Young People (FVQ_CYP_NL) [8, 15]. Parents completed the proxy versions of the PAI-CY 13–17 and evaluation form, CASP and Kidscreen. Parents also completed questions regarding sociodemographic and clinical characteristics of their child. Approximately 2 weeks after initial completion [16], participants were asked to complete a retest. Adolescents were interviewed by the same interviewer. Ophthalmic information of adolescents was retrieved from the patient files at the low vision services organizations; missing values were complemented with self-reported data from parents (*n* = 8). Visual acuity was classified using five levels based on the better seeing eye, according the criteria of the WHO [17].

### The PAI-CY 13–17

The preliminary version of the PAI-CY 13–17 contained 58 items grouped into eight domains (for descriptive purposes only, in order to provide contextual meaning) that were informed by concept-mapping workshops with end-users (see Table 2) [13]. Items were scored on a 4-point Likert scale with response options not difficult (1), slightly difficult (2), very difficult (3), and impossible (4). The response option not applicable was handled as a missing value. The items in the proxy version only differed in first or third person tense.

### Statistical analyses

As a first step in the psychometric evaluation of the PAI-CY 13–17, item reduction was performed conservatively, using lenient criteria. Items were considered for elimination if the psychometric performance was suboptimal on several criteria and if item content was already adequately represented in other items: a) Items with missing scores > 20% [8, 16] in both the adolescent and parent version and/or items with > 70% [8, 18, 19] of both adolescents and parents endorsing the first or last response category (i.e. floor or ceiling effects). b) Items showing inter-item correlations > 0.8, indicating potential redundancy. c) Suboptimal values of test-retest reliability [16] using weighted kappa and percentage agreement [20, 21]. The intraclass correlation coefficient (ICC) for sum scores of the test and retest data were calculated based on absolute agreement, with a two-way mixed-effects model.

Subsequently, adolescent-parent agreement at item level was investigated using weighted kappa and percentage agreement. The ICC for the sum scores of the adolescent and parent data were calculated based on absolute agreement, with a two-way random-effects model. Comments and suggestions evolving from the evaluation forms were used to inform decisions on item elimination or instrument adaptation.

Known-group validity on the final item-set [16] was investigated using independent samples t-tests (median split for age) and ANOVAs with a post-hoc Tukey test. Lastly, concurrent validity [16] with subscales of the Kidscreen, CASP and FVQ_CYP_NL was investigated with Spearman’s correlations. Negative correlations were expected between the PAI-CY and subscales of the Kidscreen and CASP, whereas positive correlations were expected with the FVQ_CYP_NL. Correlations were expected to be smallest between the PAI-CY and the Kidscreen and largest for the FVQ_CYP_NL.

## Results

Of an estimated 700 invited adolescents and their parents, 77 provided written informed consent and completed the first questionnaire (either the adolescent or parent version). Main reasons for non-participation were no time, not interested or adolescents not willing to participate. Sociodemographic and clinical characteristics are presented in Table 1. Complete data was obtained from 72 adolescent-parent dyads. The retest was completed by 64 parents after 28.3 ± 24.3 (range 9–121, median 18) days and 74 adolescents after 15.9 ± 7.0 (range 7–44, median 14) days.

**Table 1:** Socio-demographic and clinical characteristics of participants

Variable	All participants
Age, mean ± SD (range)	14.66 ± 1.49 (13–17)
Male gender, n (%)	49 (63.6)
Severity of VI, n (%)	
No VI: logMAR ≤0.3	25 (32.5)
Mild VI: logMAR 0.31–0.52	13 (16.9)
Moderate VI: logMAR 0.53–1.00	22 (28.6)
Severe VI: logMAR 1.01–1.30	3 (3.9)
Blind: logMAR ≥1.31 or visual field ≤10 degrees	11 (14.3)
Unknown	3 (3.9)
Comorbidity, n (%)	31 (42.5)
Parent who completed the questionnaire, n (%)	
Mother	49 (67.1)
Father	13 (17.8)
Mother and father together	8 (11.0)
Caretaker	3 (4.1)
Dutch nationality parent, n (%)	70 (95.9)
Education in years parent, mean ± SD (range)	11.53 ± 4.10 (0–16)
Financial situation parent, n (%)	
Usually enough money	38 (52.1)
Just enough money	15 (20.5)
Not enough money	8 (11.0)
No answer	12 (16.4)

*SD* standard deviation, *VI* visual impairment

Twelve items had missing responses > 20% in either the adolescent or parent version, of which eight items had > 20% missing scores in both versions (Table 2). Floor effects were found in 39 items in either the adolescent or parent version, of which 13 items displayed floor effects in both versions. Inter-item correlations of > 0.8 were found in one item pair for adolescents and seven item pairs for parents. Most items showed satisfactory test-retest reliability. For three items agreement was < 60%, whereas for eight items weighted kappa was < 0.4. ICC for the sum score on test and retest for adolescents was 0.866 (95%-confidence interval: 0.791–0.915). For parents, the ICC was 0.882 (95%-confidence interval: 0.815–0.927). With respect to adolescent-parent agreement, 34% of the items showed agreement < 60%, while 79% of the items showed weighted kappa’s ≤ 0.4. ICC between adolescent and parent scores was 0.438 (95%-confidence interval: 0.000–0.694).

**Table 2:** Distribution of responses and parameters of test-retest reliability and adolescent-parent agreement

Domain^**1**^ and item content		Missing (%)	Distribution of responses over response categories^2^ (%)				Test-retest reliability parameters		Adolescent-parent agreement	
			1	2	3	4	Agreement (%)	Weighted kappa	Agreement (%)	Weighted kappa
LT1: Doing sports	Adolescent	13.0	62.7	37.3	0.0	0.0	74.6	0.53	66.1	0.38
	Parent	7.8	38.0	52.1	9.9	0.0	64.1	0.62		
LT2: Keeping up with others during play/sports	Adolescent	3.9	68.9	27.0	4.1	0.0	66.7	0.47	42.0	0.19
	Parent	7.8	28.2	47.9	16.9	7.0	64.1	0.72		
LT3: Using social media	Adolescent	3.9	90.5	8.1	1.4	0.0	85.7	0.09	79.4	0.34
	Parent	7.8	76.1	21.1	2.8	0.0	87.1	0.61		
LT4: Playing games on computer, tablet, phone	Adolescent	15.6	83.1	16.9	0.0	0.0	80.6	0.22	74.2	0.23
	Parent	7.8	70.4	26.8	2.8	0.0	81.3	0.70		
LT5: Watching films/television	Adolescent	1.3	64.5	32.9	2.6	0.0	77.5	0.57	57.7	0.17
	Parent	6.5	48.6	44.4	6.9	0.0	75.4	0.65		
LT6: Going to a club independently^b,c^	Adolescent	46.8	90.2	4.9	0.0	4.9	83.9	0.69	68.3	0.57
	Parent	13.0	44.8	19.4	17.9	17.9	66.7	0.87		
LT7: Participating at a club	Adolescent	27.3	91.1	8.9	0.0	0.0	91.5	−0.03	54.0	0.13
	Parent	14.3	51.5	28.8	19.7	0.0	65.5	0.70		
LT8: Making music	Adolescent	51.9	70.3	27.0	2.7	0.0	75.0	0.49	61.8	0.36
	Parent	29.9	59.3	33.3	5.6	1.9	85.0	0.84		
LT9: Performing a hobby	Adolescent	33.8	76.5	21.6	2.0	0.0	79.5	0.43	64.5	0.18
	Parent	46.8	61.0	31.7	7.3	0.0	85.7	0.83		
MO1: Cycling^d,e^	Adolescent	9.1	54.0	27.1	7.1	11.4	75.8	0.78	69.2	0.74
	Parent	7.8	46.5	26.8	9.9	16.9	81.3	0.90		
MO2: Cycling to something independently^b,d,f^	Adolescent	13.0	70.1	13.4	3.0	13.4	79.0	0.90	75.4	0.70
	Parent	9.1	51.4	15.7	7.1	25.7	79.0	0.90		
MO3: Going to a friend in the neighborhood^c,e,f^	Adolescent	13.0	80.6	11.9	3.0	4.5	90.3	0.87	71.0	0.57
	Parent	7.8	54.9	21.1	15.5	8.5	74.6	0.83		
MO4: Participating in traffic independently	Adolescent	6.5	63.9	26.4	5.6	4.2	75.0	0.64	44.8	0.40
	Parent	6.5	34.7	38.9	13.9	12.5	72.3	0.85		
MO5: Estimating speeds	Adolescent	2.6	37.3	40.0	21.3	1.3	69.0	0.70	47.1	0.30
	Parent	7.8	15.5	45.1	29.6	9.9	75.0	0.70		
MO6: Using public transport independently	Adolescent	31.2	69.8	15.1	11.3	3.8	81.6	0.61	50.0	0.53
	Parent	19.5	33.9	37.1	17.7	11.3	63.3	0.82		
MO7: Learning new routes	Adolescent	3.9	68.9	24.3	5.4	1.4	68.1	0.34	47.1	0.12
	Parent	7.8	46.5	32.4	19.7	1.4	78.1	0.83		
SC1: Making contact with others	Adolescent	0.0	83.1	13.0	2.6	1.3	86.3	0.46	66.7	0.35
	Parent	6.5	61.1	30.6	8.3	0.0	72.3	0.64		
SC2: Doing activities with peers without VI^g^	Adolescent	3.9	71.6	21.6	6.8	0.0	80.9	0.70	64.7	0.37
	Parent	7.8	54.9	29.6	15.5	0.0	74.2	0.68		
SC3: Participating in group activities	Adolescent	0.0	67.5	22.1	9.1	1.3	80.3	0.55	44.9	0.16
	Parent	10.4	34.8	39.1	23.2	2.9	64.4	0.71		
SC4: Shopping with friends^a^	Adolescent	44.2	93.0	2.3	2.3	2.3	87.9	−0.05	73.0	0.46
	Parent	18.2	58.7	22.2	14.3	4.8	76.4	0.85		
SC5: Going a night out with friends	Adolescent	50.6	60.5	28.9	5.3	5.3	86.2	0.76	66.7	0.52
	Parent	28.6	36.4	34.5	18.2	10.9	69.0	0.84		
SC6: Dating^g^	Adolescent	74.0	90.0	5.0	0.0	5.0	100.0	n/a	78.6	0.00
	Parent	51.9	51.4	27.0	21.6	0.0	74.1	0.80		
SC7: Dealing with amorousness	Adolescent	58.4	96.9	3.1	0.0	0.0	100.0	n/a	83.3	0.00
	Parent	44.2	69.8	18.6	11.6	0.0	83.3	0.58		
CO1: Being able to express in words properly	Adolescent	0.0	80.5	16.9	2.6	0.0	87.7	0.69	84.7	0.61
	Parent	6.5	83.3	15.3	1.4	0.0	80.0	0.51		
CO2: Asking questions	Adolescent	2.6	80.0	16.0	4.0	0.0	77.8	0.37	70.4	0.34
	Parent	6.5	65.3	30.6	4.2	0.0	73.8	0.57		
CO3: Talking about feelings	Adolescent	14.3	60.6	28.8	10.6	0.0	67.7	0.45	47.6	0.25
	Parent	6.5	44.4	38.9	16.7	0.0	64.6	0.64		
CO4: Participating in a conversation actively	Adolescent	1.3	90.8	7.9	1.3	0.0	90.3	0.54	76.1	0.14
	Parent	6.5	79.2	18.1	2.8	0.0	84.6	0.62		
CO5: Asking help from familiar people	Adolescent	2.6	82.7	17.3	0.0	0.0	85.9	0.47	64.8	0.15
	Parent	6.5	68.1	25.0	6.9	0.0	70.8	0.37		
CO6: Asking help from unfamiliar people	Adolescent	7.8	52.1	35.2	11.3	1.4	73.1	0.46	47.8	0.25
	Parent	7.8	39.4	38.0	21.1	1.4	72.6	0.68		
CO7: Estimating emotions of others	Adolescent	1.3	56.6	34.2	7.9	1.3	74.0	0.70	63.8	0.37
	Parent	6.5	47.2	37.5	12.5	2.8	64.6	0.66		
CO8: Estimating the distance to others	Adolescent	2.6	73.3	18.7	6.7	1.3	70.0	0.58	52.9	0.09
	Parent	9.1	52.9	40.0	7.1	0.0	66.7	0.55		
CO9: Stating that you want to join in a group	Adolescent	5.2	78.1	19.2	2.7	0.0	91.0	0.73	55.9	0.36
	Parent	9.1	48.6	41.4	10.0	0.0	63.5	0.56		
CO10: Giving your opinion	Adolescent	1.3	85.5	9.2	5.3	0.0	87.5	0.60	73.2	0.09
	Parent	6.5	77.8	19.4	2.8	0.0	76.9	0.45		
CO11: Dealing with bullying	Adolescent	51.9	48.6	27.0	21.6	2.7	52.4	0.60	48.3	0.41
	Parent	28.6	34.5	43.6	21.8	0.0	59.1	0.62		
SL1: Finding the way in school	Adolescent	0.0	90.9	5.2	2.6	1.3	94.4	0.65	76.4	0.20
	Parent	6.5	80.6	18.1	1.4	0.0	85.9	0.57		
SL2: Keeping overview in class	Adolescent	1.3	81.6	14.5	2.6	1.3	87.1	0.66	50.7	0.25
	Parent	6.5	48.6	45.8	5.6	0.0	78.5	0.68		
SL3: Keeping up with classmates	Adolescent	1.3	73.7	21.1	5.3	0.0	86.1	0.66	62.8	0.26
	Parent	7.8	49.3	35.2	15.5	0.0	65.1	0.64		
SL4: Cooperating with others	Adolescent	1.3	77.6	15.8	6.6	0.0	88.4	0.83	67.6	0.37
	Parent	6.5	63.9	30.6	5.6	0.0	75.0	0.68		
SL5: Reading the slide board or schoolboard	Adolescent	6.5	50.0	26.4	15.3	8.3	65.7	0.67	41.5	0.32
	Parent	10.4	18.8	52.2	15.9	13.0	59.6	0.65		
SL6: Making homework independently	Adolescent	7.8	77.5	18.3	4.2	0.0	89.2	0.79	68.2	0.34
	Parent	6.5	63.9	30.6	2.8	2.8	76.6	0.71		
SL7: Finding information	Adolescent	2.6	74.7	20	4.0	1.3	82.1	0.44	65.2	0.14
	Parent	7.8	63.4	35.2	1.4	0.0	66.7	0.44		
SL8: Maintaining energy levels for fun activities	Adolescent	1.3	48.7	38.2	11.8	1.3	74.6	0.65	63.4	0.57
	Parent	6.5	37.5	38.9	23.6	0.0	70.8	0.75		
SL9: Choosing appropriate further education	Adolescent	54.5	71.4	11.4	14.3	2.9	73.1	0.83	54.8	0.35
	Parent	27.3	39.3	25.0	33.9	1.8	65.9	0.73		
SR1: Cooking independently	Adolescent	32.5	78.8	13.5	5.8	1.9	79.1	0.47	55.6	0.23
	Parent	24.7	48.3	29.3	19.0	3.4	87.5	0.92		
SR2: Doing the dishes	Adolescent	16.9	90.6	7.8	1.6	0.0	93.1	0.58	67.2	0.23
	Parent	7.8	64.8	29.6	5.6	0.0	83.1	0.75		
SR3: Operating devices at home	Adolescent	1.3	89.5	10.5	0.0	0.0	90.3	0.19	75.7	0.10
	Parent	7.8	73.2	21.1	5.6	0.0	79.7	0.67		
SR4: Shopping for groceries	Adolescent	15.6	76.9	10.8	7.7	4.6	79.7	0.63	69.8	0.62
	Parent	7.8	59.2	26.8	4.2	9.9	81.0	0.89		
SR5: Picking clothes independently	Adolescent	15.6	84.6	10.8	3.1	1.5	89.5	0.49	70.7	0.38
	Parent	9.1	60.0	30.0	8.6	1.4	77.4	0.78		
SR6: Brushing your teeth independently	Adolescent	1.3	96.1	2.6	1.3	0.0	94.4	0.44	88.5	0.09
	Parent	9.1	88.6	8.6	2.9	0.0	90.5	0.74		
SR7: Going to the toilet^h^	Adolescent	0.0	100.0	0.0	0.0	0.0	100.0	n/a	94.4	0.00
	Parent	6.5	94.4	4.2	1.4	0.0	93.8	0.58		
SR8: Bathing/showering independently^h^	Adolescent	0.0	97.4	2.6	0.0	0.0	100.0	1.00	95.8	0.38
	Parent	6.5	93.1	5.6	1.4	0.0	93.8	0.65		
SR9: Doing your hair	Adolescent	14.3	83.3	15.2	1.5	0.0	89.8	0.45	79.0	0.38
	Parent	7.8	77.5	19.7	2.8	0.0	81.0	0.61		
SR10: Dealing with the menstruation (girl)	Adolescent	11.1	91.7	4.2	4.2	0.0	95.8	0.88	83.3	0.67
	Parent	11.1	75.0	20.8	4.2	0.0	88.9	0.74		
AC1: Telling about your VI	Adolescent	2.6	68.0	24.0	6.7	1.3	77.5	0.64	62.8	0.31
	Parent	6.5	56.9	27.8	15.3	0.0	70.8	0.63		
AC2: Empathizing with others	Adolescent	1.3	80.3	15.8	2.6	1.3	80.6	0.44	57.7	0.20
	Parent	6.5	58.3	30.6	9.7	1.4	60.0	0.59		
AC3: Dealing with incapability	Adolescent	3.9	44.6	36.5	17.6	1.4	51.4	0.49	40.6	0.14
	Parent	6.5	30.6	55.6	13.9	0.0	61.5	0.43		
AC4: Dealing with making mistakes	Adolescent	3.9	56.8	24.3	17.6	1.4	78.9	0.78	55.7	0.38
	Parent	6.5	37.5	41.7	20.8	0.0	70.8	0.70		
FI1: Paying independently^a^	Adolescent	5.2	90.4	8.2	1.4	0.0	98.5	0.93	73.8	0.32
	Parent	13.0	70.1	19.4	7.5	3.0	81.7	0.85		

*VI* visual impairment

^1^ LT: leisure time; MO: mobility; SC: social contacts; CO: communication; SL: school; SR: self-reliance; AC: acceptance/self-consciousness; FI: finances

^2^ 1: not difficult; 2: slightly difficult; 3: very difficult; 4: impossible

^a^ item pair with inter-item correlation > 0.8 in the adolescent version

^b,c,d,e,f,g,h^ item pairs with inter-item correlation > 0.8 in the parent version

**Fig. 1:**
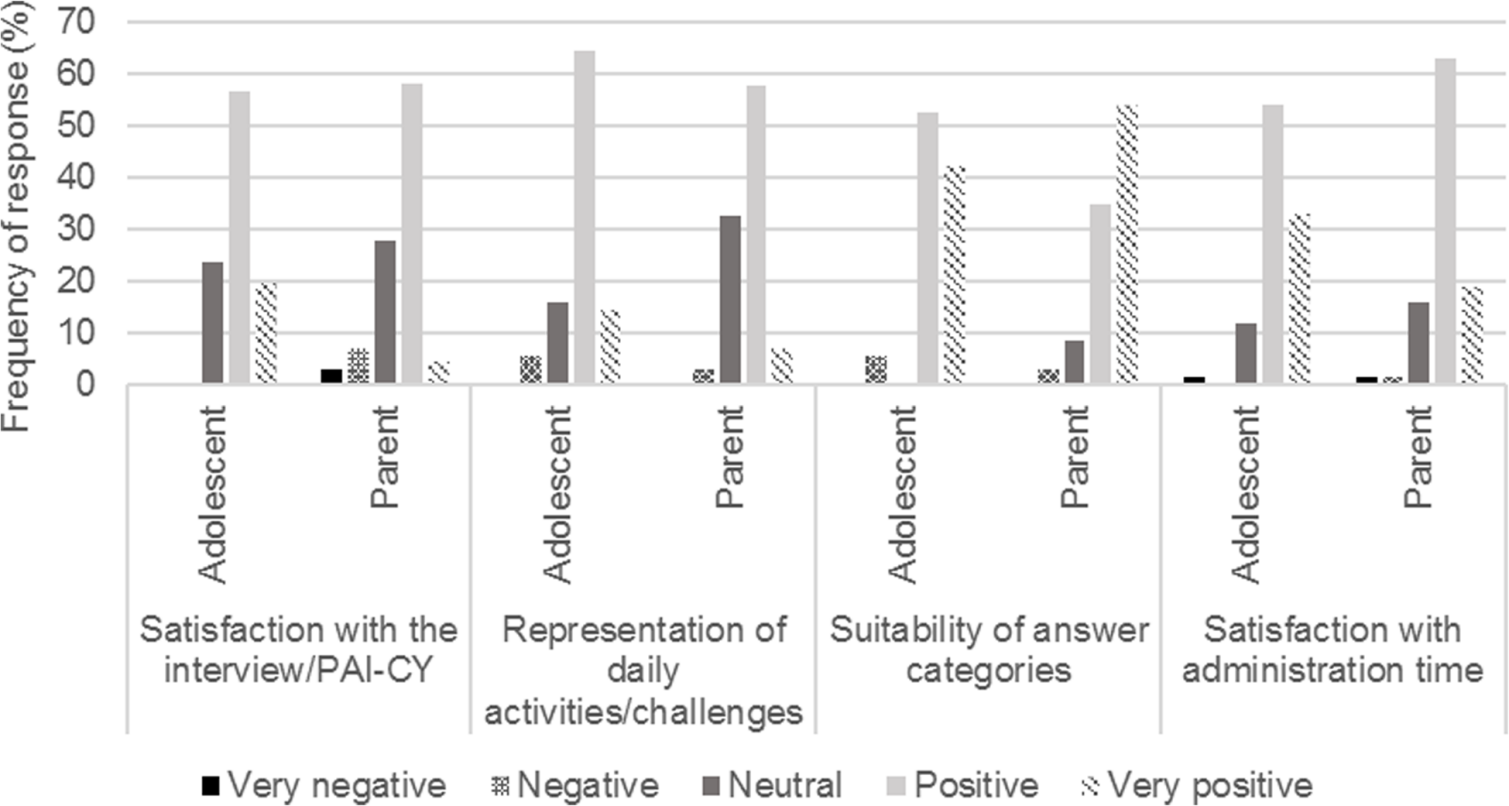
Evaluation of the PAI-CY 13–17 by adolescents and parents

The evaluation forms showed that parents’ self-reported administration time (including questions about sociodemographic and clinical characteristics) was 22 ± 14 (range 8–60, median 17) minutes. Over 90% of both adolescents and parents were neutral to very positive regarding various feasibility aspects of the PAI-CY (Fig. 1). However, comments and suggestions for improvements were made by 42 adolescents and/or parents (Table 3).

**Table 3:** Comments of > 1 respondents relevant to the PAI-CY 13–17 and suggested solutions

Comments	Suggested solutions
“Questions about make-up and personal care are missing”	Adding item: SR: paying attention to your facial care
“The textbox to give additional information should be larger”	Enlarging the textbox
“A question regarding which form of guidance is warranted is lacking”	*No adjustment – this can be filled in as response to the question clarifying needs following each domain*
“I would like to have a textbox to give additional information at the end of the questionnaire”	Adding textbox at the end of the questionnaire
“I miss questions about how siblings or the social environment deal with the disability”	Adding items: PE: difficulty of siblings regarding the visual impairment; PE: difficulty of other friends/family regarding the visual impairment
“A question regarding the needs of the parents/adolescents”	*No adjustment – this can be filled in as response to the question clarifying needs following each domain*
“Questions about self-reliance and personal care are too easy/not age-appropriate”	*No adjustment – larger samples are needed prior to deletion of these items*
“Questions about side jobs are missing”	Adding items: LT: finding and applying for a side job; LT: properly performing your side job
“A response option between slightly difficult and very difficult is lacking”	Adding the response option ‘difficult’
“I would like to have more questions regarding energy balance and fatigue”	Adding items: AC: dividing your energy over the day; AC: doing your daily activities without getting fatigued
“I miss questions about getting a driver license for a car or scooter”	Adding items: MO: finding information about the possibilities to get your driver’s license for car or scooter
“Questions regarding recognizing other people are missing”	Adding item: CO: recognizing other people
“I miss questions about school stuff outside the classroom, for example about school trips”	Adding item: SL: participating in school activities outside the regular classes
“Questions about walking in a crowded unknown environment, for example in shops, are lacking”	*No adjustment – covered by item regarding shopping*
“Questions about the difficulty of darkness or too much light are missing”	Adding item: MO: participating in traffic at night

*SR* self-reliance, *PE* parental experiences, *LT* leisure time, *AC* acceptance/self-consciousness, *MO* mobility, *CO* communication, *SL* school

From these results, it was decided to eliminate items LT8, MO2, and CO4, resulting in a PAI-CY 13–17 containing 55 items. Infrequent endorsement of the answer category ‘impossible’ motivated collapsing this category with the category ‘very difficult’. Table 4 shows expected patterns of correlations between the PAI-CY and concurrent questionnaires.

**Table 4:** Correlation coefficients of the PAI-CY 13–17 with the CASP, Kidscreen and FVQ_CYP_NL

Subscale	Adolescents	Parents
CASP total	− 0.71*	− 0.87*
CASP home	− 0.49*	− 0.80*
CASP community	− 0.60*	− 0.83*
CASP school	− 0.41*	− 0.71*
CASP living	− 0.58*	− 0.83*
Kidscreen physical	− 0.39*	− 0.58*
Kidscreen psychological	− 0.41*	− 0.47*
Kidscreen parent	− 0.28	− 0.46*
Kidscreen peers	− 0.15	− 0.44*
Kidscreen school	−0.26	− 0.29
FVQ_CYP_NL	0.68*	n/a

*n/a* not applicable, *CASP* Child and Adolescent Scale of Participation, *FVQ_CYP_NL* Dutch version of the Functional Vision Questionnaire for Children and Young People

* Significant correlation (*p* < 0.01)

**Fig. 2:**
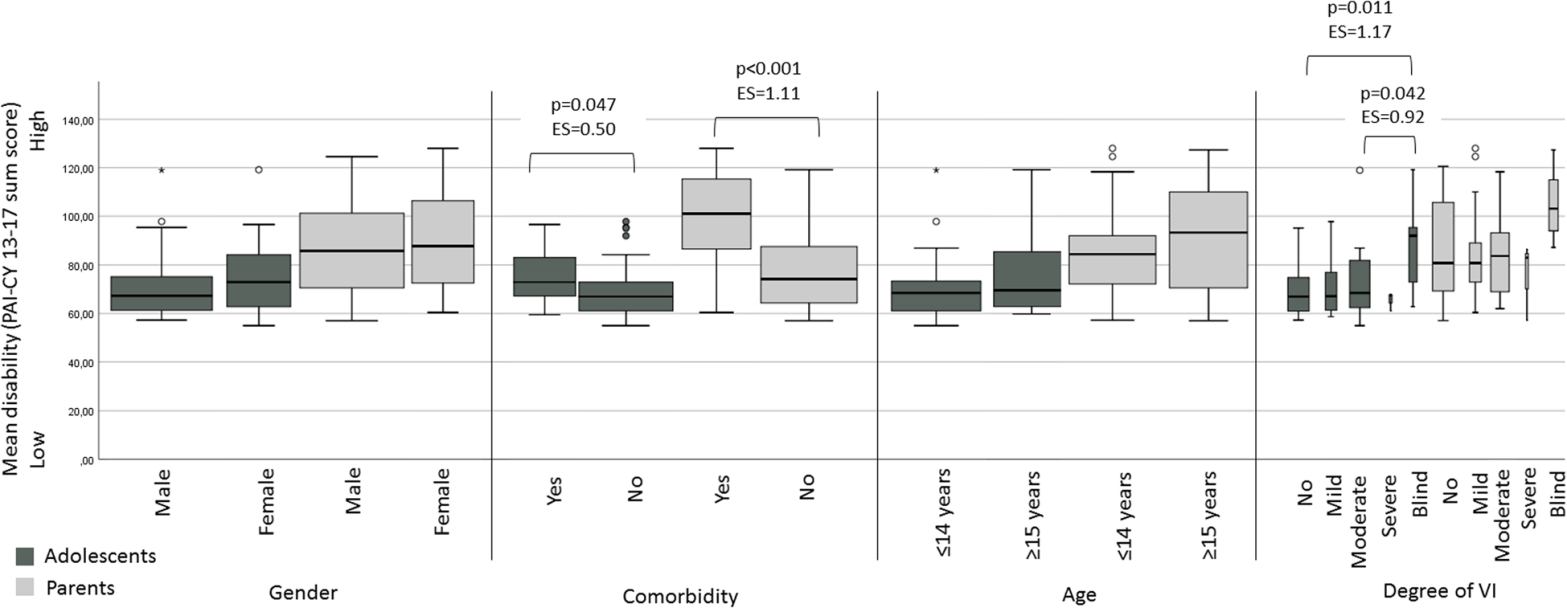
Boxplots for groups that differ on gender, comorbidity, age and degree of VI. Box width represents group sizes. ES: effect size; VI: visual impairment

Adolescents with comorbidity scored significantly worse on the PAI-CY 13–17 than adolescents without comorbidity, both in the adolescent and parent version (effect sizes were moderate to large). In the adolescent version, significantly worse scores were found for adolescents with blindness compared to those with moderate VI and no VI (large effect sizes). All other effect sizes were small (Fig. 2).

## Discussion

In this study the psychometric properties of the PAI-CY 13–17 were evaluated, a questionnaire to assess the needs of Dutch adolescents with VI and their parents. We used less stringent criteria for item elimination because of the importance of face and content validity and the small sample size. Therefore, we only eliminated three items, of which content was thought to be adequately represented in the remaining items (LT8: making music can be captured with LT9: performing a hobby, MO2: cycling to something independently with MO1: cycling and CO4: participating in a conversation actively with CO1: Being able to express in words properly). Eliminating more items while having a small sample size could be counterproductive in the long term, causing potentially informative items to be discarded before larger samples are available. Once the PAI-CY 13–17 is used in future, confirmation of the findings and the assessment of other psychometric properties should inform further item reduction, although the current number of items is in line with other age-versions of the PAI-CY and PAI-YA, containing 27–60 items [22–25]. Moreover, with larger samples item response theory modeling might be employed to make the instrument more precise and user-friendly.

Test-retest reliability was satisfactory for most items. Exclusion of participants who completed the retest > 45 days after the first questionnaire (*n* = 10) in general had a positive influence on kappa, agreement and the ICC (data not shown). Remarkably, the items with suboptimal kappa values showed adequate agreement. This phenomenon has previously been reported as the ‘paradox’ of the kappa statistic, in which low values of kappa can be found for high values of agreement [26, 27], caused by symmetrically imbalanced contingency tables [28]. For example, for item LT7: participating at a club, many adolescents opted “not difficult” on the test and retest, resulting in a high proportion of adolescents (0.98) in row 1 in the contingency table. As the proportion of respondents in row 1 increases, kappa decreases rapidly and may even fall below zero, as has happened for this item, despite excellent agreement. Therefore, agreement and weighted kappa should be re-examined with larger samples. The ICC between the sum scores of the test and retest data were high for both adolescents and parents, indicating good reliability. Adolescent-parent agreement and weighted kappa was low for many items, as was the ICC between the sum scores, indicating that adolescents and their parents have different perceptions about the difficulties adolescents experience. In general, adolescents perceive items as less difficult than parents. Notably, agreement was highest for those items taking place at home, i.e. items in the self-reliance domain. This might indicate that for activities taking place elsewhere, parents might be less aware about the difficulties experienced by their child, and therefore overestimate the difficulties experienced by adolescents. However, due to their age, adolescents also might overestimate their abilities and perceive less risks. Therefore, retrieving information from both sources is important to comprehensively assess adolescents’ needs.

It was possible to differentiate between adolescents with or without comorbidity, both with the adolescent and parent version. Furthermore, it was possible to differentiate between different degrees of VI with the adolescent version, while there was a trend with the parent version. The correlations with other instruments demonstrated concurrent validity of the PAI-CY 13–17. The relatively low correlation with subscales of the Kidscreen demonstrates that the PAI-CY 13–17 is not necessarily measuring quality of life, whereas the strong correlations with (subscales) of the CASP and the FVQ_CYP_NL confirms that the PAI-CY 13–17 rather measures a construct related to participation or functional vision. Although one can argue that the PAI-CY and FVQ_CYP_NL might be similar, the relatively strong but not perfect correlation shows that both instruments measure a similar construct, but each in its unique way.

One of the major limitations of the current study is the small sample size and the low response rate, although anticipated from previous studies involving similar populations. Main reasons for non-participation according to parents were no time, not interested or their child stating they did not want to participate. Adolescents might be reluctant to participate in scientific research, especially if they have to make an appointment and are visited by a researcher in their own homes.

The small sample size prevented factor analysis and the use of item response theory to optimize the PAI-CY 13–17. However, previous studies examining other age-versions of the PAI-CY and the PAI-YA showed the items comprised a unidimensional scale [22–24]. As such, the PAI-CY 13–17 is expected to be unidimensional as well, and we calculated sum scores for the complete scale.

The current study used a national sample, implying that the PAI-CY 13–17 should be applicable across the Dutch population. Although the PAI-CY 13–17 can be used as a template for use in other countries, cross-cultural validation is recommended for use outside the Netherlands. Recent interest to use various age-versions of the PAI-CY and the PAI-YA in Nepal and Australia, has resulted in official forward-backward translations of the instruments and manuals (available upon request from the authors) to English and Nepali, in which cultural applicability of items was also taken into account. Further research should indicate whether the PAI-CY 13–17 is indeed applicable and valid in these countries.

## Conclusions

Based on initial psychometric tests, the PAI-CY 13–17 appears to have acceptable measurement properties, although more work with larger samples is needed, for example to assess whether the items comprise a unidimensional scale. It is possible to implement the current version of the PAI-CY 13–17 in Dutch low vision services, enabling more data collection and more extensive understanding of its psychometric properties. Acceptability of the PAI-CY 13–17 to end-users should be carefully monitored, especially if the changes suggested in this study are going to be incorporated.

## Data Availability

The datasets used and/or analyzed during the current study are available from the corresponding author on reasonable request.

## Abbreviations

AC: Acceptance/self-consciousness
CASP: Child and Adolescent Scale of Participation
CO: Communication
FI: Finances
FVQ_CYP_NL: Dutch version of the Functional Vision Questionnaire for Children and Young People
ICC: Intraclass correlation coefficient
LT: Leisure time
MO: Mobility
PAI-CY: Participation and Activity Inventory for Children and Youth
PROMs: Patient-reported outcome measures
SC: Social contacts
SL: School
SR: Self-reliance
VI: Visual impairment
WHO: World Health Organization

## References

[1] Valderas JM, Alonso J (2008). Patient reported outcome measures: a model-based classification system for research and clinical practice. Quality of Life Research.

[2] Santana MJ, Feeny D (2014). Framework to assess the effects of using patient-reported outcome measures in chronic care management. Quality of Life Research.

[3] Valderas JM, Kotzeva A, Espallargues M, Guyatt G, Ferrans CE, Halyard MY (2008). The impact of measuring patient-reported outcomes in clinical practice: a systematic review of the literature. Quality of Life Research.

[4] Chen J, Ou LX, Hollis SJ (2013). A systematic review of the impact of routine collection of patient reported outcome measures on patients, providers and health organisations in an oncologic setting. BMC Health Services Research.

[5] Ravens-Sieberer U, Auquier P, Erhart M, Gosch A, Rajmil L, Bruil J (2007). The KIDSCREEN-27 quality of life measure for children and adolescents: psychometric results from a cross-cultural survey in 13 European countries. Quality of Life Research.

[6] Varni JW, Seid M, Kurtin PS (2001). PedsQL™ 4.0: reliability and validity of the pediatric quality of life inventory™ version 4.0 generic core scales in healthy and patient populations. Medical Care.

[7] Bedell G (2009). Further validation of the Child and Adolescent Scale of Participation (CASP). Developmental Neurorehabilitation.

[8] Tadic V, Cooper A, Cumberland P, Lewando-Hundt G, Rahi JS (2013). Vision-related quality of life G. Development of the functional vision questionnaire for children and young people with visual impairment: The FVQ_CYP. Ophthalmology..

[9] Khadka J, Ryan B, Margrain TH, Court H, Woodhouse JM (2010). Development of the 25-item Cardiff Visual Ability Questionnaire for Children (CVAQC). The British Journal of Ophthalmology.

[10] Birch EE, Cheng CS, Felius J (2007). Validity and reliability of the Children’s Visual Function Questionnaire (CVFQ). Journal of American Association for Pediatric Ophthalmology and Strabismus.

[11] Cochrane GM, Marella M, Keeffe JE, Lamoureux EL (2011). The Impact of Vision Impairment for Children (IVI_C): validation of a vision-specific pediatric quality-of-life questionnaire using Rasch analysis. Investigative Ophthalmology & Visual Science.

[12] Hatt SR, Leske DA, Castaneda YS, Wernimont SM, Liebermann L, Cheng-Patel CS (2019). Development of pediatric eye questionnaires for children with eye conditions. American Journal of Ophthalmology.

[13] Rainey L, Elsman EBM, van Nispen RMA, van Leeuwen LM, van Rens G (2016). Comprehending the impact of low vision on the lives of children and adolescents: A qualitative approach. Quality of Life Research.

[14] Elsman EBM, van Nispen RMA, van Rens G (2017). Feasibility of the Participation and Activity Inventory for Children and Youth (PAI-CY) and Young Adults (PAI-YA) with a visual impairment: a pilot study. Health and Quality of Life Outcomes.

[15] Elsman EB, Tadić V, Peeters CF, van Rens GH, Rahi JS, van Nispen RM (2019). Cross-cultural validation of the Functional Vision Questionnaire for Children and Young People (FVQ_CYP) with visual impairment in the Dutch population: challenges and opportunities. BMC Medical Research Methodology.

[16] De Vet HCW, Terwee CB, Mokkink LB, Knol DL (2011). Measurement in medicine: a practical guide.

[17] WHO (1994). ICD-10: International statistical classification of diseases and related health problems, 10th revision.

[18] Tadic V, Cooper A, Cumberland P, Lewando-Hundt G, Rahi JS (2016). Vision-related quality of life g. Measuring the quality of life of visually impaired children: first stage psychometric evaluation of the novel VQoL_CYP instrument. PLoS One.

[19] Pesudovs K, Burr JM, Harley C, Elliott DB (2007). The development, assessment, and selection of questionnaires. Optometry and Vision Science.

[20] Altman DG (1991). Practical statistics for medical research.

[21] Singh AS, Vik FN, Chinapaw MJM, Uijtdewilligen L, Verloigne M, Fernandez-Alvira JM (2011). Test-retest reliability and construct validity of the ENERGY-child questionnaire on energy balance-related behaviours and their potential determinants: the ENERGY-project. International Journal of Behavioral Nutrition and Physical Activity.

[22] Elsman EB, van Nispen RM, van Rens GH (2020). Psychometric evaluation of the Participation and Activity Inventory for Children and Youth (PAI-CY) 0–2 years with visual impairment. Quality of Life Research.

[23] Elsman EBM, van Nispen RMA, van Rens GH (2019). Psychometric evaluation of a new proxy-instrument to assess participation in children aged 3–6 years with visual impairment: PAI-CY 3-6. Ophthalmic and Physiological Optics.

[24] Elsman EBM, van Rens GHMB, van Nispen RMA (2018). Psychometric properties of a new intake questionnaire for visually impaired young adults: The Participation and Activity Inventory for Young Adults (PAI-YA). PLos One.

[25] Elsman EB, Peeters CF, van Nispen RM, van Rens GH (2020). Network analysis of the Participation and Activity Inventory for Children and Youth (PAI-CY) 7–12 years with visual impairment. Translational Vision Science & Technology.

[26] Feinstein AR, Cicchetti DV (1990). High agreement but Low Kappa .1. The problems of 2 paradoxes. Journal of Clinical Epidemiology.

[27] Cicchetti DV, Feinstein AR (1990). High agreement but Low Kappa .2. Resolving the Paradoxes. Journal of Clinical Epidemiology.

[28] Flight L, Julious SA (2019). The disagreeable behaviour of the kappa statistic.

